# Uptake of pharmacist recommendations by patients after discharge: Implementation study of a patient-centered medicines review service

**DOI:** 10.1186/s12877-023-03921-2

**Published:** 2023-03-29

**Authors:** Benjamin Joseph Basger, Rebekah Jane Moles, Timothy Frank Chen

**Affiliations:** 1 Discipline of Pharmacy Practice, Faculty of Medicine and Health, School of Pharmacy, The University of Sydney, Room N517, A15 Science Road, Camperdown, Sydney, NSW 2006 Australia; 2 Wolper Jewish Hospital, 8 Trelawney Street, Woollahra, Sydney, NSW 2025 Australia

**Keywords:** Polypharmacy, Patient centred care, Medicine related problems, Transitions of care, Medicine review, Inappropriate medicine use, Implementation, Deprescribing

## Abstract

**Background:**

Polypharmacy and potentially inappropriate medicine use is common in older people, resulting in harm increased by lack of patient-centred care. Hospital clinical pharmacy services may reduce such harm, particularly prevalent at transitions of care. An implementation program to achieve such services can be a complex long-term process.

**Objectives:**

To describe an implementation program and discuss its application in the development of a patient-centred discharge medicine review service; to assess service impact on older patients and their caregivers.

**Method:**

An implementation program was begun in 2006. To assess program effectiveness, 100 patients were recruited for follow-up after discharge from a private hospital between July 2019 and March 2020. There were no exclusion criteria other than age less than 65 years. Medicine review and education were provided for each patient/caregiver by a clinical pharmacist, including recommendations for future management, written in lay language. Patients were asked to consult their general practitioner to discuss those recommendations important to them. Patients were followed-up after discharge.

**Results:**

Of 368 recommendations made, 351 (95%) were actioned by patients, resulting in 284 (77% of those actioned) being implemented, and 206 regularly taken medicines (19.7 % of all regular medicines) deprescribed.

**Conclusion:**

Implementation of a patient-centred medicine review discharge service resulted in patient-reported reduction in potentially inappropriate medicine use and hospital funding of this service. This study was registered retrospectively on 12^th^ July 2022 with the ISRCTN registry, ISRCTN21156862, https://www.isrctn.com/ISRCTN21156862.

**Supplementary Information:**

The online version contains supplementary material available at 10.1186/s12877-023-03921-2.

## Introduction

Polypharmacy, defined here as the taking of five or more medicines concurrently, is associated with a high prevalence of potentially inappropriate medicine (PIM – defined in supplementary Table 1) use and occurs frequently in those aged 65 years or over [1–3]. PIM use results in poor outcomes including falls, emergency department visits, increased costs, adverse events, and functional decline [4, 5]. Deprescribing - the patient-centred, supervised process of dose reduction or cessation of PIMs [6, 7] - has been identified as part of good prescribing [8] but as limited and reactive rather than proactive, generally occurring because of an adverse event [9]. Deprescribing does not appear to be part of current hospital inpatient practice [10]. Yet the simple count of prescribed medicines at discharge has been shown to outperform complex indicators of therapy quality, such as Beers’ list 2019 [11] and STOPP criteria Version 2 [12] when identifying people at risk and predicting poor outcomes [13].

In Australia, up to 30% of hospital admissions for patients over 75 years of age have been found to be medicine-related, with up to three-quarters potentially preventable, the single most important predictor being the number of medicines taken [2]. The risk of harm and of poor adherence rises with the addition of each new medicine [14, 15], with harm described to be at epidemic proportions [16]. Transitions from hospital to primary care further increase the risk for reasons that include increased medicine sensitivity due to deconditioning and ongoing recovery from acute illness, inaccuracies in medicine reconciliation, insufficient patient education, poor communication with primary care and unexplained medicine changes [17–19]. As many as 44% of patients do not follow medicine changes initiated in hospital, continuing to take discontinued medicines, failing to implement dosage changes or to take newly prescribed medicines [20], which may themselves be potentially inappropriate [19].

While the best strategies to combat PIM use in primary care remain unclear [17, 21, 22], effective transitional pharmacist-led strategies have been described [23–28]. They have included medicine reconciliation and review in the context of multidisciplinary care, patient counselling, communication with primary care providers and post-discharge follow-up.

Although patient engagement in understanding and managing their medicines is strongly encouraged, it is uncommon [6, 29–32]. Transitional patient-centred care has been described as poorly understood and a missed opportunity for pharmacists [33], such care recognised as improving patient satisfaction and decision making and reducing adverse events and readmissions [34–38]. A paradigm shift in such care is needed [31, 39].

Australian hospital safety and quality standards state that patients and their caregivers should be actively involved in their care, and that they should receive verbal and written information in ways that are meaningful to them [40]. Patient-directed education or coaching has been shown to be the most influential component of multicomponent interventions for successful transitions [41]. However, there is limited research on the impact of pharmacy health coaching [42], or how well patient-centred care is applied to medicine management in Australian hospitals [37].

Patients have been reported to arrive at hospital taking PIMs, have PIMs commenced and be discharged on PIMs [18]. To address this problem, an implementation program for a discharge medicine review service was begun in 2006 with the development of prescribing appropriateness criteria for older Australians [43]. This criteria set was applied in a scoping study [44], which found a high incidence of PIM use at our hospital. A randomised controlled trial subsequently applied the criteria during medicine review at discharge in intervention patients, sent to patients’ general practitioners (GPs) for actioning. No significant difference in criteria-based recommendations between intervention and control groups were found at follow-up. GPs implemented a relatively low number (42%) of recommendations [45]. This led to a new intervention strategy; the patient and/or caregiver were made the driver of change in reducing their use of PIMs. A patient-centred discharge medicines review service was commenced in 2016.

This study aims to identify the processes, barriers and facilitators that influenced the implementation and intervention effectiveness of this service. For example, limited organisational resources and low leadership engagement have been identified as barriers to implementation of transitional care innovations, whereas adaptability of innovations and high perceived benefit by users identified as facilitators [39]. Implementing research into healthcare practice can be complex and unpredictable, with failure common [46–48]. A post-implementation (post hoc) study of these factors was conducted, such studies being commonly used to analyse and explain the implementation process [39, 49]. A prospective audit was conducted to determine the effectiveness of the resulting patient-centred intervention.

### Aims of the study

To describe an implementation program in the development of a patient-centred medicine review service; to assess service impact on older patients and their caregivers actioning recommendations after discharge from hospital.

### Ethics approvals

Ethics approval was obtained from the Human Research Ethics Committee of The University of Sydney for each phase of the intervention process, begun in 2006 (project numbers 2011-2015/10043, 2019/209). Approval was also obtained from the Hospitals Medical Executive Committee. Written informed consent was obtained from all individual patients or their caregivers.

## Methods

### Implementation process

Many different implementation frameworks have been developed to plan, guide, and evaluate implementation efforts [49–51]. Implementation (or process evaluation) dimensions (defined in supplementary Table 1) recommended by the Cochrane Qualitative and Implementation Methods Group [52] were identified by the authors post-intervention that determined the resulting intervention.

To gain a broad understanding of determinants of practice (that is, barriers or facilitators), a checklist resulting from a synthesis of frameworks [51] was chosen to identify determinants responsible for achieving the desired outcome. Combining different frameworks may enable a more comprehensive study [39]. Reporting was guided by the “Standards for Reporting Implementation Studies” checklist [53].

### Intervention Setting

The intervention, a prospective post-hospital audit of recommendations made to patients and/or caregivers at discharge, was carried out at a private, not-for-profit 55 bed hospital in Sydney Australia. Patients were admitted for exacerbations of chronic medical conditions such as heart failure, Parkinson’s disease, chronic obstructive pulmonary disease/asthma, degenerative spinal disease, and inflammatory bowel disease; for rehabilitation after heart, spinal, joint, gastrointestinal, breast or gynaecologic surgery, or trauma from motor vehicle accidents or falls; for palliative care due to metastatic disease; and for management of infections such as cellulitis, pneumonia or urosepsis. Chronic medical conditions and medicines were representative of older Australian community patients [45, 54]. Patients were admitted under the care of one of three geriatricians, rehabilitation specialists or one of two palliative care physicians, supported by two staff doctors. Multidisciplinary care was provided by nursing staff, physiotherapists, occupational therapists, dieticians, social workers, and a discharge planner. The clinical pharmacist (BJB) was an experienced medicines review pharmacist.

### Eligibility criteria

All patients 65 years or older were eligible. There were no other exclusion criteria. Specifically, patients were not excluded if taking less than five medicines, cognitively impaired, whose second language was English, were being discharged to residential or supportive care, lived distant from the hospital, had a terminal illness, or had vision or hearing impairment.

### Intervention

Between July 2019 and March 2020, a convenience sample of 100 patients were recruited for follow-up after discharge. Between one to four patients were discharged daily, the first alternating with the last on a non-alphabetized list being recruited daily. Where cognitive impairment was present, as determined by a Montreal Cognitive Assessment (MoCA) test [55] score of less than 26/30, or where there was language, hearing or vision difficulties, a caregiver was recruited.

Two to three days before discharge, the pharmacist explained to the patient and/or caregiver that sometimes, the benefit of taking certain medicines may be unclear, or the dose may need adjustment. A safer or cheaper medicine or even no medicine at all may be more appropriate. Permission to review their medicines, make recommendations and follow them up was sought, an information sheet provided, and a consent form signed. A medicine list would be provided that detailed the best times to take their medicines, brand names, purpose, cost considerations, relevant side effects and easy-to-understand recommendations to assist with management. Medicines were then reconciled, and reviewed utilizing validated prescribing appropriateness criteria, shown in this setting to detect approximately three quarters of all causes of medicine-related problems (MRPs) [45]. A comprehensive medicine review was conducted according to the protocol of the Pharmaceutical Society of Australia [56], including opportunities for non-pharmacologic care. Patient-directed education was provided during a discharge interview, timing facilitated by allied health staff. Patients/caregivers were encouraged to discuss with their GPs those recommendations important to them for prescription medicines, and to consider for themselves their use of non-prescription medicines. The pharmacist acted as the patient/caregivers’ advocate in proactively addressing PIM use, catering to patient/caregiver health literacy.

The discharge medicine list with recommendations and pharmacist contact details was sent separately to GPs, and where appropriate to aged care facilities, community nurses and pharmacies. Where patients had no GP, support was given finding one. Because it was necessary for all patients to have their medicines reconciled and reviewed and to receive discharge counselling, a control group was not possible. The time taken for each activity was recorded to determine the cost of the service. This included finding medical notes and walking corridors. Patients were invited to fill in a general hospital feedback form at discharge as part of standard practice.

**Fig. 1:**
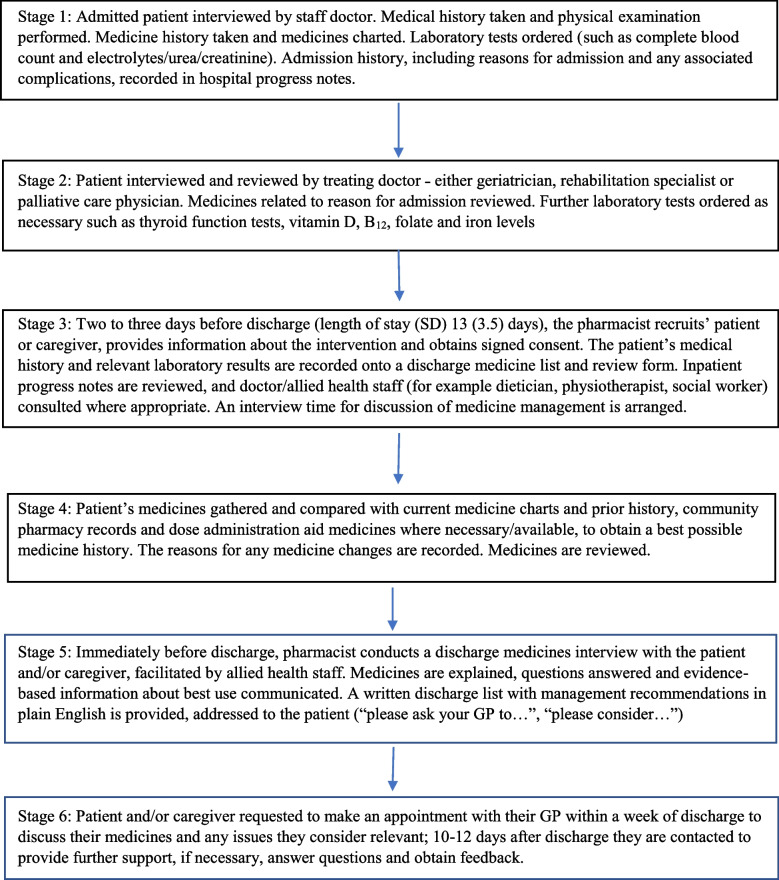
Stages of the patient journey

Ten to fourteen days after discharge, each patient or caregiver was contacted, either by phone or in person. Enquiry was made about the actioning of each recommendation, and the results including GP response recorded. Patients’ reports of changes to medicine use were accepted as truthful. Where there had been no visit to a GP or specialist doctor, support and reassurance was provided, and a repeat contact time made. The patient journey consisted of six stages (Figure 1), fitted into episodes of physiotherapy/hydrotherapy attendance, sleep, and mealtimes. Reporting followed the STROBE checklist for observational studies [57].

### Data analysis

Data were entered into Microsoft Excel (version 2203), checked for normality, and analyzed using descriptive statistics.

## Results

### Implementation

Processes and determinants identifying actions taken in the implementation of a discharge medicines review service appear in Table 1.

**Table 1:** Implementation dimensions and determinants of a discharge medicines review service ^a^ [51, 52, 60]

Timeline	Aim	Implementation dimensions ^b^	Interventions (and identification of their **determinants of practice**) ^b^	Outcomes
2006 - 2012	Identify resolve and prevent medicine-related problems (MRPs) to improve medicine management in older patients. Reduce inappropriate medicine use.	----Context National failure to reduce medicine related harm in older Australians; national review of safety and quality governance recommended development of national standards for safety and quality in health care; strong desire by hospital board and medical executive committee to improve patient care; absence of remuneration for clinical pharmacy services; known increased likelihood of MRPs occurring at transitions of care. ----Reach All patients over 65 years old taking five medicines or more. ----Implementer engagement Proactive experienced medicines-review trained pharmacist, enthusiastic and co-operative nursing, medical and administrative staff. ----Fidelity Collaboration with the University of Sydney for clinical pharmacy, research methods and service development facilitation. Participatory action research approach. ----Intervention quality A list of Australian specific prescribing appropriateness criteria to be applied to inpatients did not exist.	Private hospitals were invited to apply for a research grant by a major private health insurer, to improve any aspect of patient care. A proposal was submitted to develop and apply a list of Australian prescribing appropriateness criteria to inpatients (**payer or funder policy**). The hospital board met and determined that application of prescribing appropriateness criteria was practical (**feasibility**). University of Sydney researchers agreed to oversee the development of criteria and their application (**source of the recommendation, assistance for organisational changes**). A scoping review to examine applicability of the criteria to detect DRPs and their incidence was designed (**quality of evidence supporting the recommendation**). The hospital’s medical executive committee met and agreed that the application of such criteria was appropriate and would likely provide information necessary to progress a medicines review intervention. Cooperation was sought from attending doctors (**feasibility, implementer engagement, expected outcome, knowledge about own practice**). Ethics approval for application of criteria to patients was obtained from the Human Research Ethics Committee of the University of Sydney (**quality assurance and patient safety systems**). The director of nursing and the pharmacist liaised with all staff to explain the project and seek cooperation and feedback (**mandate, authority, accountability; team processes, implementer engagement**). The criteria had not been validated (**quality of evidence supporting the recommendation**).	A research grant of AU$30,000 was awarded by an Australian Private Healthcare Insurer to improve patient care. A list of Australian specific prescribing appropriateness criteria was developed [43] The prescribing appropriateness criteria were applied to a cohort of older inpatients. On average, each patient had seven unmet indicators of appropriateness [44] The prescribing appropriateness criteria were validated [93]
2012 -2015	Improve medicine management for older patients: Assess the impact of applying our self-developed, validated criteria-set, during medicine review at discharge, on change in the number of criteria met, health related quality of life and implementation rate of review recommendations at follow-up. Reduce inappropriate medicine use.	----Context Implementation of national safety and quality health service standards mandating systems and strategies to ensure appropriate use of medicines. Australian health service accreditation scheme introduced. Expectation by hospital board and medical executive committee of a high standard of compliance by pharmacy. Absence of remuneration for clinical pharmacy services. Increased frequency of MRPs at transitions of care. ----Dose delivered/Reach Patients over 65 years old taking 5 medicines or more, English speaking, living within a 15 km radius of the hospital, with no cognitive impairment. ----Implementer engagement Proactive experienced medicines-review trained pharmacist, enthusiastic and co-operative nursing, medical and administrative staff. ----Fidelity Collaboration with the University of Sydney for clinical pharmacy, research methods and service development facilitation. Participatory action research approach. ----Intervention quality Validated list of Australian specific prescribing appropriateness criteria. ----Adaptation Test impact via randomised controlled trial after previous scoping review.	The medical executive committee considered the results of the previous review required corrective action (**Mandate, authority, accountability; capacity to plan change, implementer engagement**). University of Sydney researchers agreed to oversee a randomised controlled trial (RCT) (**source of the recommendation, assistance for organisational changes**). The medical executive committee decided that an in-depth study to improve medicine management was needed **(Feasibility; Quality of evidence supporting the recommendation**). Attending doctors agreed (**agreement with the recommendation, implementer engagement**). Ethics approval for application of criteria to patients was obtained from the Human Research Ethics Committee of the University of Sydney (**quality assurance and patient safety systems**). The pharmacist worked part-time and researched part-time (unpaid) to perform the study (**payer or funder policy, implementation cost**). The director of nursing and the pharmacist liaised with all staff to explain the project and seek cooperation and feedback (**mandate, authority, accountability: team processes: capacity to plan change, implementer engagement**). Patients/caregivers were recruited (**Patient beliefs and knowledge**). Regular research progress reports and discussion occurred at the hospitals two-monthly Clinical Care committee meetings (**adaptation, capacity to plan change, fidelity; implementer engagement, mandate, authority and accountability; patient needs, reach, team processes).**	A randomised controlled trial was conducted [45]. There was no significant difference in the number of criteria applicable and met in intervention patients, compared to control patients. GPs implemented a relatively low rate (42%) of medicine management recommendations.
2015 - 2020	Improve medicine management for older patients: Identify resolve and prevent MRPs by changing strategy and making the patient rather than the GP or specialist doctor the driver for implementation of medicine management recommendations. Reduce inappropriate medicine use.	Context Failure to improve patient care. Absence of remuneration for clinical pharmacy services (framing the problem). ----Dose delivered/Reach Patients over 65 years old taking 5 medicines or more. Exclusion criteria present. ----Implementer engagement Proactive experienced medicine-review pharmacist, enthusiastic and co-operative nursing, medical and administrative staff. ----Fidelity Collaboration with the University of Sydney for clinical pharmacy, research methods and service development facilitation. Participatory action research approach. ----Intervention quality Validated list of Australian specific prescribing appropriateness criteria. Discharge medication form required redesigning. ----Adaptation Alter focus from the GP to the patient for implementation of recommendations. - Participant engagement/Reach Engage patients in their own medicines management. All patients over 65 years old, with no exclusion criteria.	The hospital board and medical executive supported efforts to exceed compliance with Australian accreditation standards in patient care after the results of the above RCT were considered (**mandate, authority, accountability: capacity to plan change, feasibility**). This included the establishment of paid clinical pharmacy services (**payer or funder policy, implementation cost**), resulting in positive patient feedback (**Patient needs, patients’ beliefs, and knowledge**). Pharmacist applied to the hospital for a research grant to enable payment to follow up patients after discharge; $15,000 was granted (**payer or funder policy, implementation cost**). University of Sydney researchers agreed to oversee a follow-up audit of a different strategy to improve the results of the unsuccessful RCT above (**source of the recommendation, assistance for organisational changes**). Ethics approval was obtained from the Human Research Ethics Committee of the University of Sydney (**quality assurance and patient safety systems**). The director of nursing and the pharmacist liaised with all staff to explain the project and seek cooperation (**mandate, authority, accountability: team processes: capacity to plan change, implementer engagement**). Verbal and written information about individual medicine needs and risks were made to patients in ways that were meaningful to them. The reverse side of the discharge medication list was redesigned and dedicated to medication review findings (**patient needs, patient motivation, patients’ beliefs, and knowledge**). Regular research progress reports and discussion occurred at the hospitals two-monthly Clinical Care committee meetings (**adaptation, capacity to plan change, fidelity; implementer engagement, mandate; authority and accountability; patient needs, reach, team processes).**	Current study: A cohort of older patients and/or caregivers received discharge counselling supported by written information and were asked to discuss any recommendations they thought were important with their GPs. See Results Table 2.

^a^ the three time periods described correspond to the cycles of planning, action and fact-finding characteristic of action research [60]. Cycles of planning involved researchers, the hospital’s management and administrative team, the medical executive, attending rehabilitation specialists and geriatricians, nursing, physiotherapy and occupational therapy staff and social workers. The participatory action research component contributed to the results through stakeholder inclusion and continuous two-way communication.

^b^ See table of definitions (Supplementary Table 1).

Processes of context, fidelity, implementer engagement, intervention quality and reach (definitions supplementary Table 1) appeared in each phase, as did the following determinants: feasibility; mandate, authority, and accountability; quality assurance and patient safety systems; source of the recommendation. The most commonly occurring determinants were capacity to plan change; implementer engagement; and patient needs, beliefs, knowledge, and motivation.

### Intervention

The implemented service was audited between July 2019 and March 2020. Of the 166 patients recruited, 66 were excluded; 11 were transferred to other hospitals due to the occurrence of an acute medical condition such as bleeding or chest pain, or for a procedure unavailable onsite; six left before interview; no recommendations requiring follow-up were made for 33 patients; nine patients were uncontactable after discharge; three had not seen a doctor within four weeks of discharge; three were admitted to another hospital within two weeks of discharge, and one patients family refused follow-up, leaving 100 patients.

All patients/caregivers received a discharge medicine list and review form, and all agreed to participate in a medicines discharge interview and to consider discussing those recommendations important to them with their GP. All patients were followed-up. The pharmacist did not communicate directly with GPs, nor did any GP contact the pharmacist.

Mean participant age was 83.1 years, mean total number of medicines 10.4, with a mean number of 8.9 medical conditions per patient. Of 100 patients, five took less than 5 regular medicines, 48 took five to nine regular medicines, and 47 took 10 regular medicines or more - classed as hyper polypharmacy [3]. Fifty six percent of patients were counselled in the presence of a caregiver. Of 368 recommendations made to 100 patients/caregivers, 351 (95%) were actioned, with 284 (77% of those actioned) reported to be implemented and 206 (21%) regularly taken medicines deprescribed – 141 ceased and 65 medicines reduced in dose (Table 2).

**Table 2:** Audit of implemented discharge medicine review service: study results July 2019 – March 2020

*n* = 100		
Mean age (years, SD), range	83.1 (8.1)	65 - 98
Gender (female, %)	72 (72)	
Regular medicines per patient (mean number, SD), range ^a^	9.7 (3.8)	3 - 23
“When required” medicines per patient (mean number, SD)	0.7 (0.6)	
Total number of medicines per patient (mean number, SD), range	10.4 (4.0)	3 - 25
Medical conditions per patient (mean number, SD), range ^b^	8.9 (3.2)	3 – 17
Patients counselled in presence of caregiver (number)	56	
Recommendations made (total number, per patient)	368 (3.7)	
Recommendations not actioned (total number, %)	17 (4.6)	
Recommendations actioned (total number, per patient, SD), % of total	351 (3.5) (1.5)	95.4
Recommendations not implemented ^c^ (total number, % of total)	67 (18.2)	
Recommendations implemented (total number, % of total)	284 (77.2)	
Regular medicines deprescribed ^d^ (number, % of total number), per patient	206 (21.2)	2.1
Patients who had medicine(s) deprescribed (total number)	79	
Recommended (regular) medicines commenced (number of)	13	
Patients who consulted their GP within 10 days of discharge (number of)	78	
Time taken to collect patient medical information, minutes (mean, (SD)	10.4 (2.4)	
Time taken to reconcile/review medicines, minutes (mean, (SD)	40.6 (8.6)	
Time taken to interview patient ± caregiver, minutes (mean, (SD)	12.6 (2.8)	
Patients who had cognitive impairment ^e^ (number of patients)	23	
Top five medicines deprescribed/ceased (number of patients) ^f^		
Medicines for acid related disorders – proton pump inhibitors (A02B) ^g^	27	
(Multi)Vitamins/CAMs - A11A/C/G, C10AX, B03B, M01A ^h^	23	
Mineral supplements – magnesium, calcium (A12A/C)	20	
Opioids – oxycodone ± naloxone, tapentadol, tramadol (N02A)	8	
Lipid modifying agents – statins ^i^ (C10A)	8	
Top five medicines deprescribed/reduced in dose (number of patients)		
Antiepileptics – pregabalin/gabapentin (N03A)	13	
Opioids - oxycodone ± naloxone, tapentadol, tramadol (N02A)	11	
Medicines for acid related disorders – proton pump inhibitors (A02B)	8	
Lipid modifying agents – statins (C10A)	4	
Hypnotics and sedatives – temazepam (N05C)	2	
Top ten causes of MRPs (number of times MRP occurred) ^j^		
Inappropriate medicine – not effective for the indication treated	62	
Inappropriate medicine – was not the most safe/effective	60	
Indication does not warrant medicine treatment	48	
Medicine dose too high	39	
Improvement of disease state required dosage adjustment	29	
Medicine dose too low	20	
No indication for medicine apparent	18	
Indication not treated/missing therapy	17	
Additional medicine was required to attain additive/synergistic effects	10	
No indication due to duplication	9	

^a^Regular medicine included both prescribed medicines and over the counter or complementary and alternative medicines not requiring a prescription. SD = standard deviation. Data was normally distributed. There were medicine changes during inpatient stay unrelated to pharmacist discharge recommendations (number of patients if > 1), made by treating doctors: Medicines commenced: amiodarone, amlodipine (4), docusate senna (17), doxycycline, gabapentin, irbesartan, lactulose, macrogol (9), metoclopramide (2), mirabegron, mirtazapine (3), morphine liquid, ondansetron (3), oxycodone (15), oxycodone/naloxone (11), paracetamol (21), tamsulosin/dutasteride, temazepam, tapentadol (9), vitamin D (11), warfarin. Medicines ceased: allopurinol, amitriptyline, amlodipine (4), amoxicillin (3), amoxicillin/clavulanic acid (7), atorvastatin (6), aspirin (4), bisoprolol, buprenorphine, calcium carbonate (5), candesartan, cefuroxime, ceftriaxone (2), celecoxib (2), ciprofloxacin (3), clindamycin (3), clopidogrel, clopidogrel/aspirin, colchicine (2), dexamethasone eye drops, diclofenac, digoxin (2) ,diltiazem, dithiazide, docusate senna (5), domperidone, doxepin, doxycycline (5), dutasteride/tamsulosin, empagliflozin, enalapril, enoxaparin (9), escitalopram, fenofibrate, ferrous sulphate, fish oil (2), flucloxacillin, fluticasone inhaler, folic acid (2), furosemide (3), gabapentin (2), glucosamine, heparin (2), hydrochlorothiazide, imipramine, irbesartan (3), irbesartan/hydrochlorothiazide, isosorbide, lenalidomide, lactulose, lercanidipine (3), macrogol powder (10), magnesium aspartate (5), melatonin, meloxicam (4), metformin (4), methenamine, methotrexate, metoclopramide (2), moxonidine, multivitamins (6), nebivolol, nizatidine (2), ondansetron (3), Olmesartan, omeprazole (2), oxycodone (7), oxycodone/naloxone (6), pantoprazole (2), paracetamol (5), perindopril, phentermine, phosphate (2), potassium chloride (6), prednisone (2), prazosin, pregabalin (2), ramipril (2), ranitidine (2), rasagiline, rosuvastatin (2), sacubitril/valsartan, sildenafil, simvastatin, sitagliptin, solifenacin, spironolactone (2), tadalafil, tapentadol (3), telmisartan (4), telmisartan/hydrochlorothiazide, temazepam (3), thiamine (2), tiotropium/olodaterol inhaler, tramadol, trimethoprim, trimethoprim/sulfamethoxazole, valaciclovir, vitamin C, vitamin D (10), zinc. Medicine dose increased: allopurinol, bisoprolol, furosemide (3), gabapentin (2), irbesartan, levothyroxine (3), methyldopa, mirtazapine, perindopril, potassium chloride, pregabalin, trandolapril. Medicine dose decreased: amiodarone, amlodipine, apixaban, bisoprolol, calcium carbonate, candesartan, cholestyramine, furosemide, irbesartan, lercanidipine, magnesium aspartate, metformin, metoprolol (2), potassium chloride, pregabalin (2). Medicines type changed: Warfarin to apixaban, pregabalin to gabapentin, apixaban to rivaroxaban, dipyridamole/aspirin to aspirin. Medicine doses being weaned: prednisone. Medicines not administered despite being charted: acitretin, fish oil, glucosamine, macrogol, magnesium aspartate, multivitamins, nicotinamide, terbutaline inhaler, vitamin D, vitamin C, zinc. Patients generally had no choice about these medicine changes.

^b^Classified according to the World Health Organisation International Classification of Diseases [94]. Disease/Condition classification, followed by the total number occurring, followed by the most commonly occurring in each classification: Diseases of the circulatory system (BA-BE) (191) - hypertension (63), coronary heart disease (32), atrial fibrillation (32), heart failure (21). Diseases of the musculoskeletal system (FA-FC) (128) – osteoarthritis (49), osteoporosis (34), degenerative spinal disease (31). Symptoms signs or clinical findings not elsewhere classified (MA-MH) (101) – joint pain (26), cognitive impairment (23), falls (19), incontinence (13), oedema legs (6). Endocrine nutritional or metabolic diseases (5A-5D) (83) – dyslipidaemia (55), hypothyroidism (16). Diseases of the digestive system (DA-DE) (83) – gastro-oesophageal reflux disease (62), diverticulitis (10). Diseases of the nervous system (8A-8E) (57) – neuropathic pain (22), cerebrovascular accident (12), Parkinson’s disease (9). Diseases of the genitourinary system (GA-GC) (40) – chronic kidney disease (18), benign prostatic hypertrophy (11). Mental behavioural disorders (6A-6E) (33) – depression (24), anxiety (8). Neoplasms (2A-2F) (36) – breast cancer (15).

^c^Recommendations that were understood and acknowledged by the patient/carer/family member and/or GP but not actioned.

^d^Deprescribed medicines were those reduced in dose or ceased (defined in Supplementary Table 1).

^e^Per Mini-Mental State Examination (MMSE) [55] score of less than 26/30.

^f^Classified according to the World Health Organisation Anatomical Therapeutic Chemical (ATC) classification system [95]

^g^Proton pump inhibitors (PPIs) (esomeprazole, omeprazole, pantoprazole, rabeprazole). Sixty-two patients had been prescribed PPIs.

^h^CAMs = complementary and alternative medicines, defined as non-prescription medicinal products containing ingredients such as nutritional supplements, herbal therapies and homeopathic products taken orally. Vitamins and CAMs included in descending count order (top 6), glucosamine, multivitamin preparations, vitamin D, fish oil, folic acid, and vitamin C.

^i^Atorvastatin, fluvastatin, pravastatin, simvastatin, rosuvastatin. Over half the cohort (55%) had been prescribed statins.

^j^More than one medicine related problem (MRP) may have occurred in an individual patient. MRPs classified according to a validated classification system [58].

There were 340 causes of a medicine-related problems (MRPs - 3.4 per patient), classified according to a validated system [58]. The top 10 categories represented 92% (312/340) of all causes of MRPs, the most common being: Medicine not effective for the indication treated; medicine was not the most safe/effective; and indication does not warrant medicine treatment (Table 2)

Medicines for acid-related disorders, multivitamins, complementary and alternative medicines, and mineral supplements were the most common medicines ceased. Gabapentinoids, opiates, proton pump inhibitors and statins were the most common medicines reduced in dose. The time taken to reconcile, review and interview patients/caregivers averaged 63.6 minutes/patient.

Recommendations not actioned (17 or 4.6% of the total number) occurred if patients/caregivers decided they were unimportant. Recommendations not implemented occurred because medicines were continued despite evidence provided of poor or absent effectiveness, or GPs considering recommendations unnecessary. Examples included non-discontinuation of glucosamine [59] and prescription of proton pump inhibitors despite apparent lack of indication. Oral feedback about the service from attending doctors and nursing staff, and written feedback from patients presented at patient care committee meetings, was consistently positive with respect to the quality and usefulness of the service.

Examples of medicine management recommendations made to patients appear in supplementary Table 2, according to the cause of their medicine related problem.

## Discussion

Continuing positive feedback and the results of this study resulted in our non-government, not-for-profit (private) hospital commencing and continuing to pay for a non-dispensing or cognitive pharmacy service. Facilitators influencing the implementation of transitional care innovations have been identified and include the benefits and usefulness of the innovation to healthcare providers; patient satisfaction resulting in high buy-in from healthcare providers and management; quality of information transfer; clear roles and responsibilities of key team members; support from allied health and administrative staff; and regular communication and feedback about the innovation [39]. These facilitators appear in this study.

Gaining the approval of the Hospital’s executive officers, board of management and medical committee was considered critical in legitimizing the clinical role of the pharmacist. The Hospital supported implementation from inception, providing organizational and policy support. Allied healthcare team support was also essential to facilitate implementation, contributing to the design and evaluation of the service at each stage. This has been found to make interventions more likely to be effective at ward level [60] and represented a participatory action research approach [61]. Such an approach has been used to improve care of delirium in older inpatients [62] and to address inappropriate psychotropic medicine use in residential care [63]. Staff understood that the pharmacist taking time to talk to patients/caregivers about medicines was fundamental to patient care.

Patient-centered care appeared to be of low priority in Australian hospitals [37, 64] and internationally [31, 65, 66], featuring poor delivery of information [28, 67–70]. Transition interventions involving caregivers also appeared uncommon [25, 31, 41] and often with poor pharmacist involvement [35]. Caregivers need to be recognized as partners in management to reduce communication failures and share information received by patients [32, 71, 72]. Care delivered in this study motivated patients/caregivers to become effective facilitators of medicine management change after discharge. Educating patients/caregivers facilitated crossing the primary-secondary interface, where the pharmacist was made the person for accurately determining and explaining the appropriateness of patients’ medicines and providing it in plainly written form [71]. Such a model of pharmacist care did not appear to be standard practice [73].

In a realist synthesis of pharmacist-conducted medicine reviews in discharged patients [74], factors likely to lead to beneficial outcomes were discussed. Corresponding to these factors, this study engaged healthcare professionals, patients, and caregivers; recruited patients in a trusted environment supportive of the integral role and skill of the pharmacist; established hospital organizational support; provided a pharmacist who understood the critical role of medicine review and integration with staff; and had access to comprehensive information about patients [74].

Handover at transitions of care involved transfer of responsibility to GPs. However, in this study, PIM use was identified and discussed with the patient/caregiver, who were requested to take it up with their GP if it concerned them. This differed from standard practice of pharmacists making recommendations directly to GPs. [24]. GPs then had their attention directed to PIM use by a concerned patient. This proved effective in influencing GPs decision-making behavior (the “nudge” strategy [75]) through overcoming personal cognitive biases, habits, fear of upsetting the patient, therapeutic inertia (failure to alter therapy when indicated [76]) or psychological reactance – a motivational state that affirms a person’s freedom of choice, even if opposite to a recommendation [77].

The presence of MRPs after discharge was not unusual, as hospital doctors may not review long-term medicines unrelated to the current admission, viewing it as the GPs role [78]. After discharge, the GP may assume that medicines have been evaluated and were appropriate to continue. Lack of hospital review represented a lost opportunity, as most older Australians were willing to stop one or more of their regular medicines if their GP said they could [79, 80].

### Strengths and limitations

The behavioural nudge featured in this study requires confirmation [81]. Cost of the service appeared dependent upon pharmacist time per patient. Follow-up was short, although persistence of discharge medicine changes following medicine review have been demonstrated [82]. Patients/caregivers reports of medicine changes were accepted as truthful, with no further form of validation. This study was performed in a small hospital by a single pharmacist, limiting generalisability. No clinical outcomes were reported. However, the implementation process delivered a funded service judged effective by management. There were no patient exclusion criteria other than age, adding to real-world impact.

## Conclusion

An implementation program resulted in the commencement of a paid patient-centred discharge medicine review service with an implementation rate of recommendations exceeding that of a previous effort. Failure of patient centred care appeared common in hospitals. This, combined with low rates of medicine review in those recently discharged from hospital [32], meant that the epidemic of medicine-related harm may remain undiminished.

## Supplementary Information

**Additional file 1.**

**Additional file 2.**

## Data Availability

Individual participant data is available from Dr Ben Basger at ben.basger@sydney.edu.au upon request. It consists of an Excel spreadsheet containing de-identified patient gender, age, number of medicines taken, and category of medicine related problems identified. All other data generated or analysed during this study is included in this published article.

## Abbreviations

GP: General Practitioner (family doctor)
MoCA: Montreal Cognitive Assessment Test
MRP: Medicine Related Problem
PIM: Potentially Inappropriate Medicine
STOPP: Screening Tool of Older Persons’ Prescriptions
STROBE: Strengthening the reporting of observational studies in epidemiology
