# Supplementary material for: Uptake of pharmacist recommendations by patients after discharge: Implementation study of a patient-centered medicines review service
Source: BMC Geriatr. 2023 Mar 29;23:183. doi: 10.1186/s12877-023-03921-2 (PMC10061906; doi:10.1186/s12877-023-03921-2)
Supplement: Supplementary file 1 — Additional file 1. [file 12877_2023_3921_MOESM1_ESM.docx]

**Supplementary Table 1** Definition of terms

| Term | Definition | Comments |
| --- | --- | --- |
| Adaptation ^2^ | Aspects of a program intentionally changed during delivery to enhance outcomes. | Includes factors influencing adaptation of intervention activities. |
| Adverse drug event (ADE) | A response to a medicine that is harmful, unintended and may have been avoidable [83, 84] | Adverse drug reactions (ADRs) may be regarded as a subset of ADEs. ADRs are a response that is harmful, unintended, and unavoidable [83, 84] |
| Agreement with the recommendation ^1^ | The extent to which the healthcare professionals agree with the recommendation | Attending doctors (general practitioners, rehabilitation specialists, geriatricians) accepted the quality of the evidence and the need for an intervention. |
| Assistance for organisational changes ^1^ | The extent to which external support is needed for necessary changes | External to the healthcare facility. |
| Capacity to plan change ^1^ | The extent to which healthcare professionals have the capacity to plan necessary changes | Extra resources or (re)organisation to assist with implementation may be needed. |
| Context ^2^ | Factors (such as social and political) that shape implementation |  |
| Deprescribing | The patient-centred, supervised process of dose reduction or cessation of potentially inappropriate medicines (PIMs) [6, 7]. |  |
| Determinant of practice ^1^ | Factors that might prevent or enable improvements in that practice | Such factors have also been referred to as barriers and enablers, barriers and facilitators, and problems and needs. |
| Dose delivered ^2^ | Amount of a program delivered to participants (such as frequency, duration, and intensity). |  |
| Expected outcome ^1^ | The extent to which healthcare professionals believe that the recommended intervention will lead to desired outcomes. | Audits/surveys may need to provide compelling evidence for the intervention. |
| Feasibility ^1^ | The extent to which the clinical intervention is practical. | The extent to which a new intervention can be successfully carried out within a given setting. It may involve recruitment, retention, and participation rates [85]. |
| Fidelity ^2^ | Implementation integrity, adherence, and extent to which a program is implemented as intended. | Does the program or service function as intended by the intervention developers [85]? |
| Implementation | A planned and deliberately initiated effort to put an intervention into practice [52]. | Lack of information on intervention implementation weakens internal validity and inhibits the translation and uptake of evidence by decision-makers to inform policy and practice [52]. |
| Intervention quality ^2^ | The quality of intervention resources. | For example, ability and time of paid interventionist; organisational efficiency. |
| Implementation cost | The cost impact of an implementation effort. | Includes the cost of the strategy used and the cost of the service delivery. |
| Implementation dimension ^2^ | Process evaluations that focus on aspects of implementation | Process evaluations seeks to understand the processes associated with successful knowledge translation in a particular setting; to understand the relationship between specific program elements and program outcomes [85]. |
| Implementer engagement ^2^ | Subjective staff attributes that influence program delivery. | What they think/feel about the intervention. |
| Implementation | A planned and deliberately initiated effort with the intention to put an intervention into practice. ^2^ | Implementation strategies are methods used to improve implementation of health care innovations/interventions. ^2^ |
| Knowledge about own practice ^1^ | The extent to which healthcare professionals are aware of their own practice in relationship to the recommended practice. | Audit/surveys may need to provide compelling evidence for the intervention. |
| Mandate, authority, accountability ^1^ | The mandate, authority, and accountability for making necessary changes | Relates to personnel responsible for the organisational changes necessary to facilitate implementation. |
| Medicine related problem (MRP) | Actual or potential negative outcomes resulting from the way in which medicines are used or underused [86] | MRPs consist of two categories (1) medicine treatment goals are not reached (the medicine does not work), and (2) medicine treatment causes an undesirable effect (the medicine causes harm). A medicine whose dose is too low or too high, for example, would be a *cause* of a MRP [58]. |
| Medicine review | A patient-focused collaborative service that critically reviews the use of all prescribed and non-prescribed medicines to optimise understanding and minimize medicine-related problems [87]. |  |
| Participant engagement ^2^ | Participants interaction with or receptivity to a program. | What participants think or how they feel about the intervention. |
| Patients’ beliefs and knowledge ^1^ | Healthcare professionals’ ability to inform or teach patients necessary knowledge and skills. | Is the pharmacist able to effectively provide accurate medicines information in ways that are meaningful to the patient? |
| Patient-centred care | Care that is respectful and responsive to the wishes of the patient [88]. |  |
| Patient motivation ^1^ | The healthcare professional’s ability to motivate patients for change | Patient behaviours/beliefs may mean that they are not motivated to change or are unaware of a potential need for change. Includes allied health input. |
| Patient needs ^1^ | Real or perceived needs and demands of the patient. | Does the pharmacist’s perception correspond with actual patient needs? |
| Payer or funder policy ^1^ | The extent to which payer or funder policies may affect implementation. | Negotiation with payers/funders to support change may be necessary. Alternatively, lack of such a policy may not affect an implementation strategy. |
| Potentially inappropriate medicines (PIMs) | Medicines used in older people that have no clear evidence-based indication, carry a substantially higher risk of adverse effects compared to use in younger people or to medicines of equal or greater efficacy, are not cost-effective or are prescribed despite non-medicine measures being more appropriate [89-91]. | Potentially inappropriate prescribing has been described as the presence of over-prescribing (that is, no clear indication), under-prescribing (the omission or under-dosing of potentially beneficial medicines) and mis-prescribing (the prescription of a medicine that significantly increases the danger of an adverse drug event, such as dose too high) [92]. |
| Process evaluation | Investigates the activities and internal dynamics of an intervention during its implementation to determine how well it operates [52]. | Process evaluations focus on aspects of implementation such as reach, fidelity and dose delivered, as defined here. They aid in understanding the relationship between specific program elements and outcomes [85]. |
| Quality assurance and patient safety systems ^1^ | The extent to which existing quality assurance or patient safety systems facilitate adherence to the recommended intervention | Implementation strategies need to comply with patient safety systems. |
| Quality of evidence supporting the recommendation ^1^ | How confident we are in the estimate of effects. | What is the quality of evidence supporting the recommendation to implement an intervention? |
| Reach ^2^ | Degree to which target group participates. | Includes factors motivating patients to participate. Also known as *penetration* – the share of eligible service recipients who use the service. |
| Source of the recommendation ^1^ | The organisation(s) and people from which the determinant of practice originated | Are the organisations(s)/people credible? |
| Team processes ^1^ | The extent to which professional teams have the skills needed to adhere and interact to facilitate improvement. | Teams consisted of nursing staff, physiotherapists, social workers, occupational therapists, and dieticians. |

^1^ Determinant of practice [51].

^2^ Implementation dimension [52, 85].
