# Supplementary material for: Uptake of pharmacist recommendations by patients after discharge: Implementation study of a patient-centered medicines review service
Source: BMC Geriatr. 2023 Mar 29;23:183. doi: 10.1186/s12877-023-03921-2 (PMC10061906; doi:10.1186/s12877-023-03921-2)
Supplement: Supplementary file 2 — Additional file 2. [file 12877_2023_3921_MOESM2_ESM.docx]

**Supplementary Table 2** Selected pharmacist recommendations according to cause of medicine-related problem (MRP)

| Cause of MRP [58] | Medicine and dose | Specific written recommendation (in addition to verbal explanations, often with a caregiver) | Outcomes |
| --- | --- | --- | --- |
| Inappropriate medicine – not effective for the indication treated | Solifenacin 10 mg at night | This medicine appeared to have no effect on reducing your night-time urinary frequency. Please ask your general practitioner (GP) to cease this medicine and consider trialing another (such as mirabegron 25 mg at night). | Solifenacin ceased. Patient preferred no alternate medicine. |
|  | Levodopa/benserazide 100/25 mg twice daily | Was commenced as a therapeutic trial for gait disturbance after your stroke some 3-4 months ago. It appeared to have had little if any effect. However, it can cause side effects like dizziness on standing and sleep disturbance. Please consider asking your GP to cease it. | GP referred patient to neurologist, who ceased it. |
| Inappropriate medicine – was not the most safe/effective | Pantoprazole 40 mg daily | This medicine was commenced 7 years ago. Unless stomach acid attacks most days (causing indigestion), please consider taking pantoprazole only when it does, as stomach acid is otherwise beneficial. | Regular use ceased after consulting GP. |
|  | Calcium carbonate 600 mg daily | Calcium tablets are large to swallow and may cause side effects like constipation and bloating. Their absorption is reduced by the stomach acid (omeprazole) tablets you are taking. Please consider replacing them with increased dietary calcium – see sheet provided. | Replaced by improved diet after discussion with family caregivers. |
| Indication does not warrant medicine treatment | Anastrozole 1 mg daily | Has been taken for about 12 years. It decreases bone mineral density and may increase risk of fractures - a concern as you have osteoporosis. Please ask your GP if you still need to take it. | GP referred patient to oncologist, who said to continue. |
|  | Rosuvastatin 10 mg daily | Please ask your GP why you need to take this medicine, as you appear to have no other risk factors for heart attack or stroke other than raised cholesterol, commenced prior to your large weight loss. | GP ceased rosuvastatin. |
| Medicine dose too high | Duloxetine 60 mg daily | You are taking 60 mg daily, and a maximum dose of 30 mg a day is recommended for someone with your reduced level of kidney function (GFR 28 ml/min). Please discuss with your GP. | GP reduced dose to 30 mg daily |
|  | Metformin 500 mg three times daily | Your blood sugars averaged 6 mmol/L at 6 am and 7.1 mmol/L during the day. It appears that your dose could be reduced to ease the number of medicines you take. Please discuss with your GP. | GP altered to one extended-release tablet at night. |
| Improvement of disease state required dosage adjustment | Irbesartan 75 mg daily | We recorded systolic (the upper number of) blood pressures mostly between 95-110 mm, which appeared to be quite low. Please ask your GP if you should continue taking irbesartan (Avapro/Karvea) | GP ceased irbesartan |
|  | Tapentadol SR 50 mg twice daily | This medicine may cause concentration and coordination problems, unsteadiness and falls, and daytime sleepiness. Your dose should be reduced slowly as appropriate. Please discuss with GP. | Dose reduced to one tablet daily. |
| Medicine dose too low | Allopurinol 100 mg daily | Your dose appears to be too low to work. Target uric acid blood level is less than 0.36 mmol/L, and yours was 0.63 mmol/L. Please ask your GP to increase your dose slowly until this target is reached. | GP agreed and began to increase dose. |
|  | Amlodipine 5 mg daily | We recorded blood pressures ranging from 135/60 – 145/70. These appear to be above target. Please ask your GP if your dose needs increasing. | GP decided to continue at this dose. |
| No indication for medicine apparent | Magnesium aspartate 500 mg daily | Please consider ceasing this medicine, as your blood magnesium levels are normal, and the only reason to use it is if they are low. | Patient chose to continue. |
|  | Sucralfate 1 g twice daily | Is used to coat the base of stomach ulcers. Since you appear to have no ulcers, please ask your GP why you need to take this medicine | Ceased by gastroenterologist. |
| Indication not treated/missing therapy | Amlodipine 5 mg twice daily | Your amlodipine (Norvasc/Nordip/Amlo) blood pressure medicine had been ceased after your spinal operation. However, we have now recorded blood pressures ranging from 135/65 – 155/80. Please ask your GP if you should re-start it. | GP told patient that blood pressure was high due to pain and to wait. |
|  | Metformin 500 mg twice daily | We recorded average blood sugars of 8.4 mmol/L (6 am) and 12.2 mmol/L during the day, which were above target levels. Your metformin had been stopped due to an episode of diarrhea. Please ask your GP if you could re-commence slowly. | Blood sugars were normal when GP checked, so metformin not restarted |
| Additional medicine was required to attain an additive/synergistic effect | Ibuprofen 400 mg twice daily | Despite use of long and short acting oxycodone and pregabalin, your pain after spinal surgery appears significant. Please consider adding ibuprofen, two tablets twice daily, as you appear to have no other medical conditions with which it may interact – discuss with your GP. | Patient commenced ibuprofen and informed GP. |
|  | Candesartan 16 mg daily | The majority of your systolic (upper measurement of) blood pressures ranged from 155 - 165 mm. Please ask your GP if you require an additional medicine, as measurements less than 140 mm, or lower if tolerated, are preferred. | GP checked and was happy with the blood pressure he measured. |
| No indication due to duplication | Rabeprazole 20 mg morning, pantoprazole 40 mg night | Please ask your GP to cease one of the two medicines that stop your stomach making acid. They both do the same thing. Please ask your GP if you can take the remaining one only when acid attacks (causing indigestion), as stomach acid is otherwise beneficial. | GP ceased rabeprazole, agreed to use when needed. |
|  | Gliclazide 80 mg, ½ morning, metformin 850 mg night, sitagliptin 50 mg night | Your doses of gliclazide and metformin could safely be doubled, which may then make sitagliptin (and its associated cost) redundant. Sitagliptin appears to be less effective than gliclazide and metformin, and your late afternoon blood sugars were above target at 12 – 14 mmol/L. Please discuss with your GP. | GP preferred no changes. |
